# Effects of lifestyle intervention using patient-centered cognitive behavioral therapy among patients with cardio-metabolic syndrome: a randomized, controlled trial

**DOI:** 10.1186/s12872-016-0398-9

**Published:** 2016-11-18

**Authors:** Ying Zhang, Songli Mei, Rui Yang, Ling Chen, Hang Gao, Li Li

**Affiliations:** 1 Department of Children and Adolescent Health Care, School of Public Health, Jilin University, #1163 Xinmin Street, Changchun, 130021 People’s Republic of China; 2 Department of Education, The First Affiliated Hospital, Jinzhou Medical University, Jinzhou, China; 3 Department of Internal Medicine, The First Affiliated Hospital, Jinzhou Medical University, Jinzhou, China; 4 Faculty of Humanity Management, Jinzhou Medical University, Jinzhou, China

**Keywords:** China, Patient-centered, Cognitive behavioral therapy, Cardio-metabolic syndrome, Randomised controlled trial

## Abstract

**Background:**

Cardio-metabolic syndrome (CMS) is a highly prevalent condition. There is an urgent need to identify effective and integrated multi-disciplinary approaches that can reduce risk factors for CMS.

**Methods:**

Sixty-two patients with a history of CMS were randomized 1:1 into two groups: a standard information -only group (control), or a self-regulated lifestyle waist circumference (patient-centered cognitive behavioral therapy) intervention group. A pretest and posttest, controlled, experimental design was used. Outcomes were measured at the baseline (week 0) and at the end of intervention (week 12). Comparisons were drawn between groups and over time.

**Results:**

The mean (standard deviation) age of the subjects was 48.6 (5.8) years ranging from 32 to 63, and 56.9% of the participants were female. Both groups showed no significant differences in Demographic variables and the metabolic syndrome indicators at baseline. While the control group only showed modest improvement after 12 weeks, compared to baseline, the intervention group demonstrated significant improvement from baseline. This study controlled for patients’ demographics and baseline characteristics when assessing the effects of intervention. After adjusting for age, education and baseline level, the experimental group and the control group were statistically significant different in the following post-treatment outcomes: WC (F = 35.96, *P* < 0.001), TG (F = 18.93, *P* < 0.001), RSBP (F = 33.89, *P* < 0.001) and SF-36(F = 157.93, *P* < 0.001). The results showed patients’ age and education were not strong predictors of patients’ outcome (including WC, TG, RSBP and SF-36).

**Conclusions:**

Lifestyle intervention on patient-centered cognitive behavioral therapy can improve the physical and mental health conditions among individuals reporting a history of cardio-metabolic syndrome, and possibly provided preliminary benefits for the treatment of CMS.

**Trial registration:**

Chinese Clinical Trial Register #, ChiCTR15006148.

## Background

Associated with the rapid economic growth, life style changes, and the aging population, cardio-metabolic syndrome (CMS) is becoming increasingly prevalent in China [1, 2] and worldwide [3]. CMS, also called the “deadly quartet”, is characterized by the presence of central obesity (also known abdominal or visceral obesity), dyslipidemia, diabetes, and hypertension. Every condition is also a risk factor for cardiovascular disease(CVD) and associated with low quality of life [4, 5]. Central obesity is the typical representation [6]. Most risk factors of CMS including overweight, no exercise, irregular sleep, unhealthy diet, and smoking,also related to the level of psychological stress [7] and the extent of morbidity [8].

Developing effective interventions to decrease the risk of CMS is critical for public health. Unhealthy lifestyle is one of the most significant public health problems in the 21st century [9, 10]. Developing good behavioral habits and maintaining balanced psychological state are necessary for people’s overall health [11]. According to the World Health Organization, an 'unhealthy lifestyle' is defined as the failure to achieve the minimum recommended physical fitness exercises (i.e., for adults, 150 min of moderate aerobic exercise or 75 min of vigorous aerobic exercise per week, or an equivalent combination). However, at least 60% of the global population fail to meet the standard [12].

To achieve optimum effectiveness, cognitive behavioral therapies are designed to concentrate on changing the patient’s negative cognitive habits and emotion, and restructuring the healthy thinking patterns and promoting positive feelings. The ultimate goal is to change the patients’ lifestyle and health-related behaviors. In addition, this therapy lets the participants to be their own therapists [13] in order to effectively control their lifestyle and prevent chronic disease [14]. Lifestyle modification based on cognitive behavior therapy is an essential component of the comprehensive approach to manage the CMS. Effective lifestyle intervention to increase health benefits is considerably important, and cognitive behavioral procedures represent additional nonsurgical intervention for reducing the risk of CMS.

To address the poor adherence [15], the longer-term therapy generally requires a partnership with the patients [16]. A growing body of literature explores using patient-centeredness and shared clinical decision-making to improve treatment outcomes for people with chronic diseases [17]. “Patient-centeredness” puts the patient’s needs the foremost when addressing their disease issues. “Patient-centered” communication is a behavior intervention approach that elicits, respects and incorporates patients’ wishes and allows active patient participation [18]. Shifts from the biomedical, paternalistic model to more patient-centeredness suggests the efforts should focus on delivering belief that incorporates patients’ needs, preference, goals, will, and paying attention to their decisions when providing reasonable medical treatment [19, 20].

Behavioral health presents psychology and its sister professions with new opportunities for training, research, and practice [21]. In the last few years’ studies have demonstrated beneficial effects of interdiscipinary approach combining exercise and healthy diet for the treatment of obesity [22] and MS [23], lifestyle interventions can be observed from a combination approach, such as exercise and patient-centered cognitive behavioral therapy in both depression and type 2 diabetes outcomes [24], and low-energy-density dietary counseling and cognitive behavioral therapy for obesity and binge eating disorder [25].

We suggest that patient-centered cognitive behavioral therapy emphasizes integrated care, putting patients first, improving patients’ cognitive, emotional and behaviorial styles,and further accept and conform healthy lifestyles. In the present study, 127 adults with CMS were randomized to further study the effectiveness of lifestyle intervention offered by a 12-week patient-centered cognitive behavioral therapy on health indicators and quality of life among participants with CMS. The lifestyle intervention of patient-centered cognitive behavioral therapy (PC-CBT) constructs incorporated shared clinical decision, supervised exercise sessions, dietary education and advice, advice for the smoking cessation, and the promotion of lifestyle behavioral change using Skinner’s behavior intensified techniques. We hypothesized that the intervention should decrease the risk of CMS, and increase the patients’ quality of life when compared with the advice-only control approach.

## Methods

### Participants

At enrollment, all volunteers agreed in written form to provide their data anonymously and receive necessary treatment provided by their doctors. Eligible patients were at least 18 years of age, and metabolically affected by the CMS. The cardio-metabolic syndrome was defined using the 2005 IDF (international diabetes foundation) recommendations: waist circumference (primarily based on the epidemiological data of Shanghai and Hong Kong) [26]) ≥ 90 cm for males and ≥ 80 cm for females, fasting serum-triglyceride levels of ≥ 1.7 mmol/L, blood pressure ≥ 130/85. All participants were able to read and understand Chinese, which was the language of the questionnaire. Patients who could not adhere to treatment during the intervention period because of their physical conditions, mental state or other reasons determined by a physician were excluded. The research was approved by the Institutional Review Board (IRB) of the Jinzhou medical university affiliated hospital. All ongoing and related trials of this intervention are registered.

### Main outcome measurement

Socio-demographic characteristics concerning age, gender, employment status, marital status, level of education were obtained from all patients, and concurrent medication uses were also recorded by the researcher. Life style information before and after the intervention was also collected. Outcome measurements included in this study were: waist circumference, fasting serum-triglycerides levels, resting systolic blood pressure, and health-related quality of life. The TG levels were collected from the participant’s latest three months clinic records. The waist circumference (WC) was measured in a standing position at the midpoint between the lowest rib and the iliac crest at the late exhalation.

The quality of life was based on a 36-item short form of health survey (SF-36). SF-36 includes 8 scales and 36 items: physical functioning (PF, 10 items), physical role functioning (RP, 4 items), bodily pain (BP, 2 items), general health perceptions (GH, 5 items), mental health (MH, 5 items), social functioning (SF, 2 items), emotional role functioning- (RE, 3 items), vitality (VT, 4 items), and health transition (HT, 1 item). Physical and mental health summary scores are derived from the eight scales. Higher percentile scores represent a better quality of life.

#### Procedures and setting

Volunteers were recruited from Jinzhou, a regional city in Northeastern China. The study protocol was approved by the Human Research Ethics Committee at the first affiliated hospital of Jinzhou Medical University (KY 201407) on December 18th 2014. Data were collected between April 2015 and November 2015. Recruitment was conducted through the distribution of flyers in the community calling for volunteers to participate in the PC-CBT lifestyle intervention trial which was aimed at changing people’s lifestyle habits to reduce CMS risk factors and improve their health condition All participants understood the study and gave their written informed consent before he/she was enrolled in the lifestyle workshop curriculum. Then participants were randomly assigned to receive the lifestyle intervention using PC-CBT constructs or “consultation only” (control). The individual participant’s assignments were sealed in serially numbered envelopes. The whole laboratory analyses were conducted by technicians blinded to treatment assignment. Participants and investigator were not blinded because of the nature of the treatment.

### Intervention

Twenty-four workshops were conducted for the intervention group. In contrast to the control group, volunteers in the “PC-CBT” group received several workshops developed for lifestyle change based on patients-centered cognitive behavioral therapy. The workshop was offered 2 days per week for a group of minimum five patients. It consists of eight modules, each lasts 90–120 min. The content of the workshop is largely based on global guidelines and the PC-CBT constructs but some modules include condition-specific contents. The workshop contents are shown in Table 1.

**Table 1:** Components of PC-CBT lifestyle intervention

Workshop curriculum modules	Subjects
Module 1: break ice	Welcome, introduce each other, subjects overview
Module 2: interaction with each other deeply	Acquaintance each other, talking about their own families, work if have one, tailoring communication to preferences, needs and values, discussing differences
Module 3: talking about health	Communication about CMS, challenge and opportunities, sharing of illness related fears and concerns, risk assessment
Module 4: talking about lifestyle	Methods in health regulation, exercise, eating habit, and so on
Module 5: methods in psychology adjustment	CBT, behavioral reinforcement, meditation, stress management, and abdominal breathing
Module 6: patient-centered	Shared-clinical decision-making scheme, self-deterrnination theory, participant attitude, awareness of being responsible for their own health
Module 7: viable lifestyle change program	Reasonable diet and exercise, don’t stay up late, eliminate smoking, limit alcohol consumption, night-time snack eating habits
Module 8: close	Planning of personal goal, close

Note: *CMS* cardio-metabolic syndrome, *PC-CBT* patient-centered cognitive behavioral therapy

A psychologist and an internal medicine physician led the workshop. For some modules an old CMS patient with a chronic condition and a clinician specialized in geriatrics were invited as experts. Group leaders established good relationships with the patients. Based on a standardized interview questionnaire [27], patients were asked about their opinions regarding lifestyle intervention options such as being conscious about their health, or being persistent on exercise. To attain feasible lifestyle goals, we considered patients’ specific circumstances, individual preferences, needs and values, and ensured that patients’ values directed all clinical decisions [28]. Therapists applied shared decision-making according to patients specific condition and value system. Both therapists and patients were involved in making the goals for the lifestyle change, and therapists also educated the participants on CMS as a chronic disease, the need for self-determination program, and the importance of cooperation with clinicians. The objective of the patient-centered discussion is to let the patient understand how the lifestyle change is generally taking place and accept the concept of healthy behavior according to individual’s condition.

The behavioral reinforcement component of the intervention was integrated into the healthy lifestyle session and was based on Watson's operational behavior reflection theory. Meditation was also required as described in the broad spectrum cognitive-behavioral therapy [29]. Concurrent psychological cognitive-behavioral therapy such as stress management training, and abdominal breathing were also integrated into these sessions. The aim was to modify and maintain participants’ healthy behavior and being more conscious about their health. Such approach has demonstrated efficacy in clinical populations previously [30, 31].

Lifestyle modification program was individualized after considering the attitude of the participants [32]. Participants were empowered to independently develop a lifestyle change program based on shared decision-making process. Patients were also guided and encouraged to adopt and maintain 150 min of medium exercise per week, a 200–300 kcal reduction in daily dietary calories [33], do not stay up late, quit smoking and limit alcohol consumption. Patients were guided and encouraged to develop new recipes to increase the intake of high quality food that are rich in protein, potassium and calcium. Furthermore, patients were encouraged to reduce fat intake and consume more fruit and vegetables, and alter night-time snack eating habits. Exercise activities such as walking, where the patients could be accompanied by a relative or friend, were especially encouraged. Individualized recommendations on lifestyle change were made to be feasible and practical.

### Control group

Participants allocated to the advice-only control group received a letter explaining their group allocation and basic lifestyle advice and general information about CMS risk factors. They were sent weekly text messages about the standard care for CMS. There were no in person contacts with the control group during the study other than the scheduled measurements.

### Statistical analysis

Analysis of the data from this study was performed using SPSS 21.0 statistical software (SPSS, IBM, Lmd, Beijing, China). We attempted to establish the clinical significance of the lifestyle intervention on PC-CBT constructs in the treatment of cardio-metabolic syndrome using a pre- and post-test equivalent group design. All analyses presented here are based on data from subjects who completed the 12-weeks study. To validate these results, additional calculations were performed by replacing all missing data with baseline data, according to the “baseline observation carried forward” method [34].

Outcome measurements were assessed at baseline before randomization (week 0) and at the end of the intervention (week 12). Mean and standard deviations are used to describe the baseline characteristics and post-treatment status for continuous variables. Statistical analysis of the outcome differences between the groups, after 12 weeks, was performed using a Student's t test. Categorical variables are presented as frequencies and percentages. The differences were considered statistically significant when the p value is < 0.05 (two-tailed). General linear regression was used to analyze the treatment effect, adjusting for differences on baseline characteristics (age, marriage status, education level, and employment state) and baseline levels of WC, TG, RSBP and SF-36 total.

## Results

### Study population and patients characteristics

**Fig. 1:**
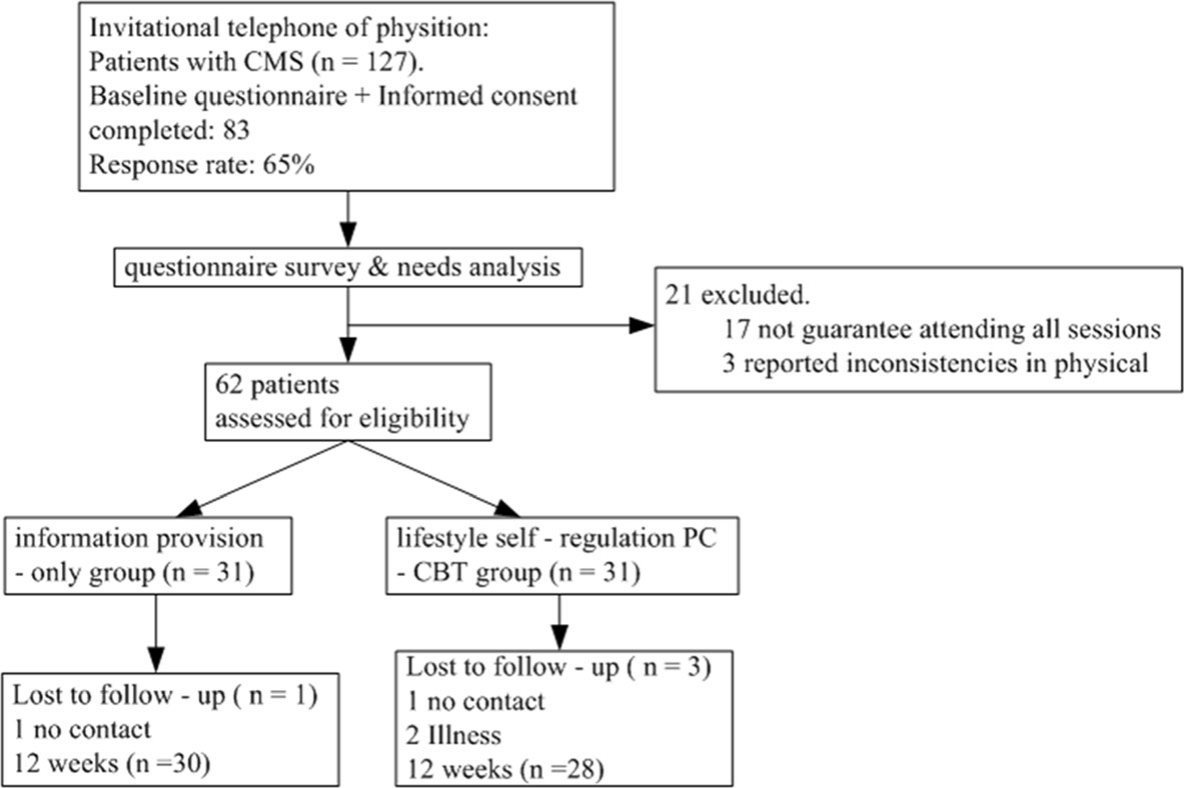
Detailed flow of participants through the study

A total of 127 patients with CMS were identified, and 83(65%) were qualified for study criteria. Of those, 62 expressed interest in participating along with the survey and were randomized into 2 groups: the 12-week PC-CBT, and standard lifestyle consultation-only. During the study, 58 patients (28 and 30 in the PC-CBT or control groups, respectively) had complete follow-up (93.5%). Figure 1 shows the patients enrollment and follow-up throughout the study.

The mean age of the CMS patients was 48.6 years (SD = 5.8; range = 32–63) with 56.9% of the patients above 50 years. The majority of patients were female (56.9%). Most people were married and living together with partners (63.8%). One third of the participants (34.5%) held a degree or post graduate qualification and 39% were employed full-time. The average duration of CMS from diagnosis was 9.6 (SD = 3.2) years. Clinically, most patients were considered to be obese (63.8%) and 55.2% had hypertension.

### SF-36 reliability analysis

The scores obtained from the patients completed the SF-36 survey were converted to a percentile score. In this study, SF-36 had a cronbach alpha value of 0.899. The alpha coefficients for the two sub-scales (Physical Health and Mental Health) were 0.845 and 0.836, respectively. These values conform to the measurement requirements.

### Baseline characteristics

In order to confirm that the 58 participants who completed this study in the intervention and control groups were comparable, we compared their lifestyle factors, biomarker levels, SF-36 and relevant comorbidities at baseline (Table 2). No significant differences were found for WC and TG, and SF-36 characteristics between two groups (except a borderline significant difference in RSBP value (*p* = 0.03) which was higher in the control group).

**Table 2:** Raw data by group and *P*-value at baseline point and the end of intervention

	Baseline characteristics				The end of intervention			
	(week 0)				(week 12)			
	All subjects	Control	Intervention	*P*-value	Control	Intervention	mean diff.(95% CI)	*P*-value
Not smoking (N, %)		18(60)	16(57.1)		22(73.3)	24(85.7)		
Saturated fat(≤10% of total energy)(N, %)		14(46.7)	13(46.4)		18(60)	23(82.1)		
Fruit and vegetables(≥400 g per day)(N, %)		9(30)	10(35.7)		13(43.3)	22(78.6)		
Physical activity(≥150 min per week)(N, %)		11(36.7)	11(39.3)		16(53.3)	20(71.4)		
WC total (cm)	94.2 ± 3.2	94.9 ± 2.8	93.4 ± 3.4	0.071	94.1 ± 2.8	86.0 ± 2.4	−8.1 (−9.5, −6.7)	.000***
WC in men	—	96 ± 2.4	94.6 ± 3.9	0.215	95.2 ± 2.4	87.3 ± 2.3	−7.9 (−9.6, −6.2)	.000***
WC in women	—	93 ± 2.5	92 ± 2.3	0.212	92.5 ± 2.8	84.5 ± 1.6	−8.0 (−9.9, −6.2)	.000***
Cardio-metabolic								
TG (mmol/L)	2.0 ± 0.4	2.1 ± 0.2	1.9 ± 0.5	0.127	1.9 ± 0.2	1.1 ± 0.3	−0.8 (−0.9, −0.7)	.000***
RSBP (mmHg)	140.5 ± 3.4	140.0 ± 3.2	141.5 ± 3.3	0.032	137.9 ± 3.2	134.5 ± 3.7	−3.4 (−5.2, −1.6)	.000***
Quality of life								
SF-36 total	419.3 ± 112.4	409.8 ± 87.5	414.6 ± 116.8	0.764	530.1 ± 95.6	637.2 ± 58.2	107.1 (65.6, 148.1)	.000***
MH	48.1 ± 17.3	49.9 ± 17.6	46.1 ± 17.0	0.416	63.9 ± 16.6	69.9 ± 13.0	6.0 (−1.9, 13.9)	0.133
RE	46.6 ± 25.7	43.3 ± 27.9	50.0 ± 23.2	0.328	62.2 ± 24.4	77.4 ± 15.8	15.2 (4.3, 26.1)	.007**
PF	48.8 ± 12.6	49.7 ± 12.7	47.9 ± 12.6	0.588	61.5 ± 13.3	79.0 ± 13.8	17.4 (10.3, 24.6)	.000***
RP	47.8 ± 27.0	47.5 ± 28.1	48.2 ± 26.3	0.921	61.7 ± 26.9	81.2 ± 14.6	19.6 (8.2, 30.9)	.001**
BP	55.2 ± 20.0	55.2 ± 22.0	55.1 ± 17.9	0.974	68.6 ± 20.7	82.0 ± 12.8	13.3 (4.3, 22.3)	.000***
SF	60.3 ± 15.4	57.5 ± 14.2	63.4 ± 16.3	0.146	70.8 ± 13.7	87.1 ± 8.7	16.2 (10.2, 22.2)	.000***
VT	57.7 ± 18.9	61.7 ± 18.0	53.4 ± 19.2	0.096	76.7 ± 15.1	80.4 ± 11.5	3.7 (−3.4, 10.8)	0.303
GH	54.8 ± 19.2	58.8 ± 16.7	50.5 ± 21.1	0.101	64.7 ± 18.2	80.4 ± 10.8	15.7 (7.9, 23.5)	.000***
Obese (N, %)								
hypertension (N, %)		21 (70)	16 (57.1)		21 (70)	9 (32.1)		
hypertriglyceridemia		13 (43.3)	19 (67.9)		10 (33.3)	2 (7.1)		
(N, %)		4 (13.3)	3 (10.7)		2 (6.7)	0 (0)		

Note: Continuous variables are summarized by mean ± SD. Categorical variables are reported as N (%).*** significantly different (*P* < 0.001). ** significantly different (*P* < 0.01)

*Abbreviations*: *WC* waist circumference, *TG* fasting serum-triglycerides, *RSBP* resting systolic blood tension, *SF* social functioning, *MH* mental health, *RE* role-emotional, *PF* physical functioning, *RP* role –physical, *BP* bodily pain, *VT* vitality, *GH* general health

### Comparisons of post-test indicators in control and PC-CBT groups

Table 2 also shows the proportion of patients achieved lifestyle targets and their CMS information before and after the intervention in both intervention and control groups. Participants in the intervention group achieved smoking cessation, diet and physical activity targets. Mental Health and Vitality in the health related life quality changes over time were not significantly different between groups (*p* = .133 & .303). Comparing to control group, there was a lower decrease in the levels of CMS biomarkers and also a marked increase in certain health related life quality (physical functioning; role –physical; bodily pain; general health; social functioning; role-emotional; health transition) in the intervention group.

### The effect of PC-CBT lifestyle intervention on the cardio-metabolic syndrome

We adjusted patients’ age, education, and baseline waist circumference, blood pressure, fasting serum-triglyceride levels and the SF-36 scores in GLM analysis.

The results showed that age and education were not strong predictors of patients’ outcome (including WC, TG, RSBP and SF-36). After adjusting for age, education and baseline level, the experimental group and the control group were statistically significant different in the following post-treatment outcomes: WC (F = 35.96, *P* < 0.001), TG (F = 18.93, *P* < 0.001), RSBP (F = 33.89, *P* < 0.001) and SF-36(F = 157.93, *P* < 0.001) (See Table 3).

**Table 3:** The post – intervention analyses based on GLM(*n* = 58)

Outcome variable	Test of between-subjects effects		Parameter estimates		
				95% CI	
	F	*P-Value*	B	Lower	Upper
WC total ^a^	35.960	0.000	0.535	0.357	0.714
TG (mmol/L) ^b^	18.925	0.000	0.378	0.204	0.552
RSBP (mmHg) ^c^	33.888	0.000	0.647	0.424	0.869
SF-36 total ^d^	157.932	0.000	0.607	0.510	0.703

Note: Age, education were examined separately and were not significant

^a^: Independent variable: pre WC

^b^: Independent variable: pre TG

^c^: Independent variable: pre RSBP

^d^: Independent variable: pre SF-36 total

*GLM* general linear model

*WC* waist circumference

*TG* fasting serum-triglycerides

*RSBP* resting systolic blood tension

*SF* social functioning

**Fig. 2:**
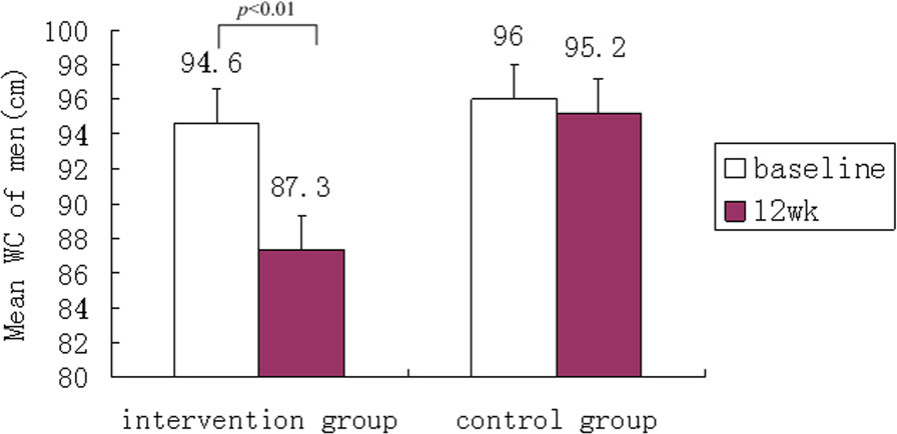
Mean Waist circumference (WC) of men between two groups

**Fig. 3:**
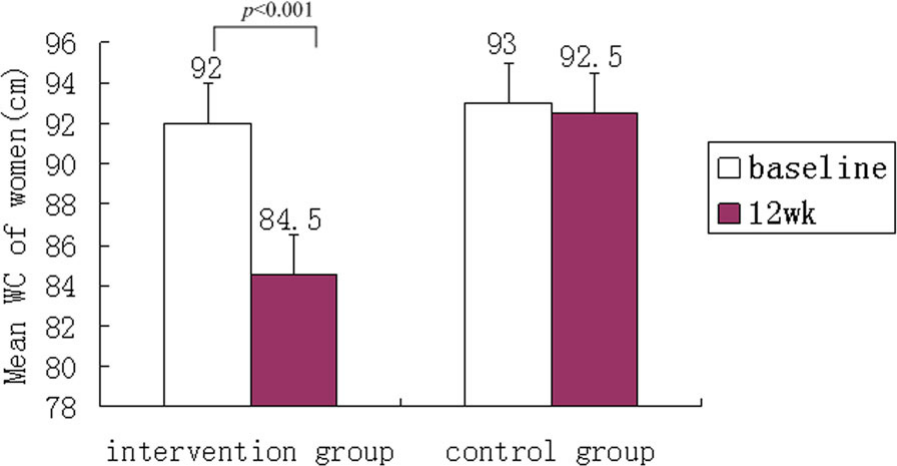
Mean Waist circumference (WC) of women between two groups

Post-test value shows that the waist circumference in female remains higher than the high risk threshold (>80). Changes of waist circumferences from baseline and by gender after the lifestyle intervention are shown in Fig. 2 and Fig. 3. Compared to the controlled group, the intervention group showed significant decreases in WC (−7.9, *p* < 0.001 in men and −8.0, *p* < 0.001 in women).

## Discussion

This study evaluated the effect of PC-CBT lifestyle intervention on cardio-metabolic syndrome. Patients randomized to PC-CBT lifestyle intervention received self-involved management, were introduced to routine exercise and healthier diet, combined with psychological behavioral change program for 12 weeks. The intervention was well received by the patients, represented by high compliance rate (90.6%).

Individuals with obesity who participated in the program of health dietary and regular exercise had reduced CMS risk over time, demonstrating the potential value of lifestyle intervention [35–37]. The critical effect of psychological processes on the success of lifestyle change suggests that new psychological and patient-centeredness approach should be adopted in the lifestyle intervention, to help CMS patients develop their own favorite healthy lifestyle, and change their old and unhealthy behavior. Combined together, these approaches provide more hope for an effective treatment of CMS [38].

The results of this study show that PC-CBT lifestyle intervention leads to remarkable reductions in waist circumference, fasting serum-triglycerides levels, resting systolic blood tension, and improved quality of life when compared to the control group. The magnitude of these changes was significant and may be adequate to provide clinical benefits. Waist circumference and CMS biomarker level reductions after PC-CBT lifestyle intervention are typically greater than those without [39, 40]. A possible explanation of the mechanism responsible for this change is shared-clinical decision making, where the responsibility for behavioral changes is transferred from counselors to the patient.

Similarly, an effective CMS treatment strategy should include the engagement of patients in determining healthy lifestyles and maintaining the results also requires specific strategies [41]. Patient-centered intervention has been considered to be an indispensable element of healthcare [42]. The concept of patient-centered approach comprehensively combines the cognitive, emotional and behavioral techniques. We think the intervention that played a significant role is those connected with patient-centeredness. Good relationship is the basis of good clinical decision. The treatment plan also considers individual’s personality, self-perception, and health-centered consciousness. The other explanation may be: shared-decision, self-determination and the use of behavior intensifying techniques, which enhance the sense of self-control and self-efficacy in patients, which further enhanced inner motivation to keep a healthy lifestyle [43].

Participants were self-reported at baseline as CMS. We selected general indicators such as waist circumference (central obesity), resting systolic blood tension, fasting serum-triglycerides, as these are especially relevant to the psycho-social aspects of CMS [44–46]. And we considered the psycho-social factors and life qualities’ subjective evaluation in CMS patients.

We used the conceptual framework and methodology described by the 2005 IDF (international diabetes foundation) recommendations. For a Chinese patient to be classified as “recovered”, they must meet the following criteria: waist circumference < 90 cm for males and < 80 cm for females. If a person experienced clinically significant change, but failed to meet the established criteria for normal function, they are considered “improved but not recovered” [47]. Our study shows that the mean waist circumference of women post-intervention was 84.5 ± 1.6 cm while the pre- intervention mean was 92.0 ± 2.3 cm, which is considered “improved but not recovered”.

For patients with non-communicable disease, the usual treatment is pharmacological intervention. However, evidences have shown that these patients should take more responsibility for their health [48], and be more conscious about their healthy lifestyle, which can be crucial for the success of their disease management. Behavior patterns represent the single most prominent domain of influence in public health [49]. However, unhealthy behavior is often enjoyable, hard to resist. Furthermore, there is a weak connection between various types of beneficial behavioral practices. For example, a man who often exercises may still smoke. Patient-centered self-management is the centerpiece of behavioral intervention programs.

Psychology, self-monitoring and lifestyle behavioral interventions rarely alleviate CMS in the long-term, with many patients regaining these symptoms after therapy [50]. It is very likely that such interventions come from consultants’ suggestions rather than from patients’ inner motivation. And it produces a rapid effect on the cardio-metabolic syndrome that is gradually lost in the longer term. There is a consensus to control these chronic diseases by primarily adjusting patients’ behavior and lifestyle, rather than using a pure biomedical approach [51].

For patients, learning the knowledge of good lifestyle practices is not enough to ensure that positive choices are made. Our intervention was developed based on the premise that a therapy approach based on self-motivation, cognitive changes that leads to behavior change and the practice of a “healthy lifestyle”, could offer more sustainable long-term benefit.

This patient-centered intervention, based on the shared-decision-making and self-management, emphasizes the importance of flexibility in the way that physicians structure the decision-making process so that individual differences on their goals and preferences can be respected. This research showed PC-CBT lifestyle intervention reduced CMS symptoms as we hypothesized. Changing the health behaviors of chronic patients is difficult, it is therefore necessary to conduct more patient-centered therapy.

### Limitations

A significant association between self-determination behavior and healthy lifestyle on the reduction of CMS risk was consistently found in this study. One limitation of our study was the reliance on the self-reported quantitative index, which is a subjective evaluation about their quality of life. Furthermore, our sample size was determined by the recruitment conditions in this specific patient population, the sample size was small and participants randomized for the intervention research.

So the results may not be generalizable to other CMS patients. While cognitive behavioral treatment and patients-centeredness are time-consuming, this study demonstrates that PC-CBT lifestyle intervention has a positive effect in promoting the CMS patients’ physical, psychological, and social functions within a short time. Longer-term studies are required to monitor if these changes are maintained.

## Conclusion

It confirmed that active training in healthy psychological and behavioral intensification is more effective than passive provision of guidelines. Such interventions should include patients-centeredness, emotional attention, cognitive adjustment, perceived social support and behavior intensification techniques. Continued clinical follow up may be required to further investigate for long-term maintenance in individuals attempting psychological and behavioral lifestyle change. The improved combination of psychological and behavioral approaches with counseling should be further investigated in a home-based model.

## Abbreviations

BP: Bodily pain
CMS: Cardio-metabolic syndrome
CVD: Cardiovascular disease
GH: General health
IDF: International diabetes foundation
IRB: Institutional Review Board
MH: Mental health
PC-CBT: Patient-centered cognitive behavioral therapy
PF: Physical functioning
RE: Role-emotional
RP: Role –physical
RSBP: Resting systolic blood tension
SF: Social functioning
TG: Fasting serum-triglycerides
VT: Vitality
WC: Waist circumference
